# In vivo measurement of pH and CO_2_ levels in the uterus of sows through the estrous cycle and after insemination

**DOI:** 10.1038/s41598-021-82620-7

**Published:** 2021-02-04

**Authors:** Octavio López-Albors, Pedro José Llamas-López, Joaquín Ángel Ortuño, Rafael Latorre, Francisco Alberto García-Vázquez

**Affiliations:** 1 Department of Anatomy and Comparative Pathology, University of Murcia, 30100 Murcia, Spain; 2 International Excellence Campus for Higher Education and Research (Campus Mare Nostrum), Murcia, Spain; 3 Department of Physiology, Faculty of Veterinary Science, University of Murcia, 30100 Murcia, Spain; 4 Department of Analytical Chemistry, Faculty of Chemistry, University of Murcia, 30100 Murcia, Spain; 5 Institute for Biomedical Research of Murcia, IMIB-Arrixaca, Murcia, Spain

**Keywords:** Reproductive biology, Animal physiology

## Abstract

The pH–CO_2_–HCO_3_^−^ system is a ubiquitous biological regulator with important functional implications for reproduction. Knowledge of the physiological values of its components is relevant for reproductive biology and the optimization of Assisted Reproductive Technologies (ARTs). However, in situ measurements of these parameters in the uterus are scarce or null. This study describes a non-invasive method for in situ time-lapse recording of pH and CO_2_ within the uterus of non-anesthetized sows. Animals were at three different reproductive conditions, estrous with no insemination and two hours after insemination, and diestrous. From pH and CO_2_ data, HCO_3_^−^ concentration was estimated. The non-invasive approach to the porcine uterus with novel optical probes allowed the obtaining of in situ physiological values of pH, CO_2_, and HCO_3_^−^. Variable oscillatory patterns of pH, CO_2_ and HCO_3_^−^ were found independently of the estrous condition. Insemination did not immediately change the levels of uterine pH, CO_2_ (%) and HCO_3_^−^ concentration, but all the values were affected by the estrous cycle decreasing significantly at diestrous condition. This study contributes to a better understanding of the in vivo regulation of the pH-CO_2_-HCO_3_^−^ system in the uterus and may help to optimize the protocols of sperm treatment for in vitro fertilization.

## Introduction

Carbon dioxide (CO_2_) and H_2_O are the most common end products of the energy catabolic pathways in living organisms. In multicellular organisms, carbonic anhydrase mediates the reaction between these two molecules to deliver carbonic acid (H_2_CO_3_), which rapidly dissociates into a H^+^ and a bicarbonate ion (HCO_3_^−^). The resulting equilibrium between CO_2_, H^+^ and HCO_3_^−^ plays a pivotal role in the regulation of many relevant biological processes such as breathing, diuresis or reproduction^1,2^.

The success of fertilization starts with sperm migration within an adequate microenvironment of the female genital tract. Both, H^+^ and HCO_3_^−^ have a pivotal role in regulating sperm fertilization capacity, which is progressively achieved along their trajectory from the epididymis to the fertilization site in the oviduct through a gradient of increased pH and HCO_3_^−^ concentrations^3–5^. Both components are directly involved in many biochemical reactions resulting in the so called `sperm capacitation`, a process necessary for fertilization^6,7^. HCO_3_^−^ and pH play also an important role for embryo transport, development and implantation^2,8^. The production and secretion of HCO_3_^−^ and H^+^ in the uterus, although not fully elucidated, includes different exchangers^9,10^ which are sensitive to sex-steroids hormones^8,10–13^.

Reference in vivo levels of pH, CO_2_ and HCO_3_^−^ in the uterus and oviduct of several mammals (rabbit, monkey, human, bovine, equine or porcine) have been obtained during different stages of the estrous cycle (see Table 1), while other values were obtained from oviductal or uterine fluid samples^14,15^. pH is the best characterized parameter. Uterus and oviduct pH vary between species (range from 6.83 to 7.35 and 6.7–8.3, respectively) and is influenced by the stage of the estrous cycle (Table 1). Contrastingly, no HCO_3_^−^ values have been recorded directly from the uterus, so the available data were obtained from oviductal fluid, either directly or from samples^14–16^. Likewise, very few studies and with minimal explicit information have been aimed at determining CO_2_ tension (pCO_2_) within the uterus or oviduct^14,16^. In the porcine species, pH values in the oviduct are very variable (ranged from 6.7 to 8.3) depending on the study and the stage of the cycle^15,17,18^, while HCO_3_^−^ levels of 10.0–33.1 mMol were estimated in samples of oviductal fluid retrieved under surgical conditions^15^. In the uterus, pH (6.98) was only measured in a preliminary study in a series of 6 gilts^17^, with no mention to CO_2_ or HCO_3_- values.

**Table 1:** Summary of the studies measuring pH, CO_2_ and/or HCO_3_^−^ levels directly in the female genital tract (uterus and/or oviduct) of different species.

Species	Organ	Estrous cycle	pH	HCO_3_^−^ (mMol)	pCO_2_	References
Rabbit	Oviduct	–	7.94	–	–	^24^
Primate (*Macaca mulatta)*	Oviduct	Follicular phase	7.1–7.3	35*	–	^16^
		Luteal phase	7.5–8.0	90*	–	
		Menstrual cycle	–	–	89 Torr (46–143)	
Bovine	Uterus	Day 0 estrous cycle	7.22	–	–	^23^
		Day 1–6 estrous cycle	7.28–7.35	–	–	
	Uterus	Day 0 estrous cycle (estrous)	6.85–6.89	–	–	^20,21^
		Day 7 estrous cycle (diestrous)	7.03–7.15	–	–	
	Uterus	–	7.00–7.05	–	–	^25^
	Oviduct	Day 0–6 estrous cycle	7.41–7.60	–	–	^19^
	Uterus	Day 6–14 estrous cycle	6.96–7.21	–	–	
Equine	Uterus	Estrous	~ 6.83–6.97	–	–	^22^
Porcine	Oviduct	Estrous	7.02	–	–	^17^
	Uterus	Estrous	6.98	–	–	
	Oviduct	Pre-ovulatory phase	7.76–7.96	–	–	^18^
		Post-ovulatory phase	7.37–7.40	–	–	
	Oviduct	Estrous	6.7–8.3	–	–	^15^

*Calculated from CO_2_ and pH values.

The characterization of physicochemical parameters in vivo is always a challenge. The use of medication to suppress any pain or stress caused by the surgical approach or during the recording of data always has an impact on the physiological condition. Hugentobler et al.^19^ compared the influence of the anesthetic protocol on oviductal and uterine pH in cows. When intravenous anesthesia (thiopentone) was compared with inhalatory anesthesia (halothane) oviduct and blood pH was reduced, whereas uterus pH increased. For this reason, the most reliable results in this type of approaches are those with minimum or complete absence of pre-medication. This has been achieved in cows^20,21^ and mares^22^, where a non-invasive external approach to the uterine lumen via the cervix allowed direct pH measurements. However, until now a similar approach to the uterus has not been tried yet in pigs.

In most previous studies the in vivo measurement of uterine pH was carried out with potentiometric devices. For this, miniaturized pH glass electrodes either as a single unit or in combination with a reference electrode were extensively used^15–25^. Similarly, for pCO_2_ measurements a pH electrode was converted to a Severinghaus type electrode covered with a Teflon membrane^16^. In recent years, optical sensors have become a convenient alternative in several areas of research, including biological systems and organisms. Different principles and working mechanisms are used depending on the analyte to be measured^26^ and for optical pH and CO_2_ measurements, a combination of different fluorescent dyes which detect intensity changes in the time domain are used. Although optical based probes have been used to assess in vivo oxygen levels within the female reproductive tract of humans^27^ and pigs^28^, to our knowledge, this technology has never been used in reproductive organs for pH and CO_2_ measurement.

The porcine species is being widely used as an experimental animal model in biomedicine because of its similarities with humans in many aspects (i.e. genomics and immunology system)^29,30^. Despite the differences observed in the female genital tract when compared with humans, porcine arises as a good alternative to other commonly used animal models such as rodents. In addition, to produce these porcine biomodels in a standardized scale, Assisted Reproductive Technologies (ARTs) are necessary (reviewed by^31,32^). However, porcine in vitro fertilization (IVF) is far from being as efficient as in other species as rodents or bovine^32^. The main problem is polyspermy (more than one spermatozoon penetrates the oocyte), which has been related with the protocols of gamete preparation, and the medium and ambient conditions stablished during the co-incubation of gametes, which makes the zygote non-viable. Thus, when the ambient parameters mimic the physiological values, as it was recently demonstrated by adjusting the oxygen level for IVF and embryo culture^28^, porcine ARTs yield better results. With regard to the pH–CO_2_–HCO_3_^−^ levels, the sperm cells and oocytes used for IVF are directly immersed in a culture media whose values for these parameters might not be similar to the physiological. This is mainly due to the absence of appropriate knowledge about the real figures existing in the living animal. The same problem is found for the protocols of sperm preparation, since no consideration is taken towards the transitional period confronted by the sperm cells in their passage throughout the uterus. Given that there is an almost complete lack of knowledge about the regulatory mechanisms of uterine pH^19^, whether the uterine ambient, and particularly its luminal pH–CO_2_–HCO_3_^−^ levels are modulated by the presence of sperm is a topic which deserves further attention.

From the above information we hypothesized that the measurement of pH–CO_2_–HCO_3_^−^ levels in the porcine uterus by minimally invasive methods and no medication will give reference results (physiological), which should help to better understand the physiological ambient of the uterus at different stages of the estrous cycle, and when measurements are taken after insemination, how these parameters are modulated by the presence of ejaculate. For this purpose we aimed the estimation of pH, CO_2_ and HCO_3_^−^ under three different conditions, (i) sows in estrous before artificial insemination [E( −)AI]; (ii) sows in estrous 2 h after AI [E( +)AI] ; and (iii) sows in diestrous (non-estrous stage, [NE]). Ultimately, these results could be used to better adjust the in vitro protocols to increase the efficiency of porcine ARTs.

## Results

### Attempts to monitoring pH and CO_2_ levels in the uterine cavity: validation of the technique

In general terms the technique used to approach the uterine lumen with the probes was successful. After implanting the insemination catheter in the cervix, the progress of the inner cannula up to 16 cm towards the uterine cavity helped to widen the distal portion of the cervical canal. Then, the successive approaches with the endovascular catheter -with temperature probe inside-, the pH and CO_2_ probes were successful in nearly all the animals. One subjective but unequivocal sign of having reached the uterine cavity was the hand-feeling of a smooth progression of the probe after having overpassed the inner most cervical cushion. However, in two sows at NE condition, the deepest part of the cervical canal did not allow for further progress of the pH and CO_2_ probes. On the other hand, CO_2_ data from one pig at the three experimental situations were dismissed because inaccurate readings of the sensor, and from another pig at E( +)AI because of some bleeding was observed. Hence, the approach was successful in 68 out of 72 attempts (94.4%, pH and CO_2_ probes in 12 animals at three different conditions) and the recording of data in 64 occasions (88.9%). The average insertion length of the pH and CO_2_ probes was 64.2 ± 12.6 cm and 62.0 ± 11.5 cm, respectively. The average temperature of the uterus was 38.2 ± 0.3ºC.

### pH measurements within the uterus during estrous (before and after insemination) and diestrous stages

**Figure 1:**
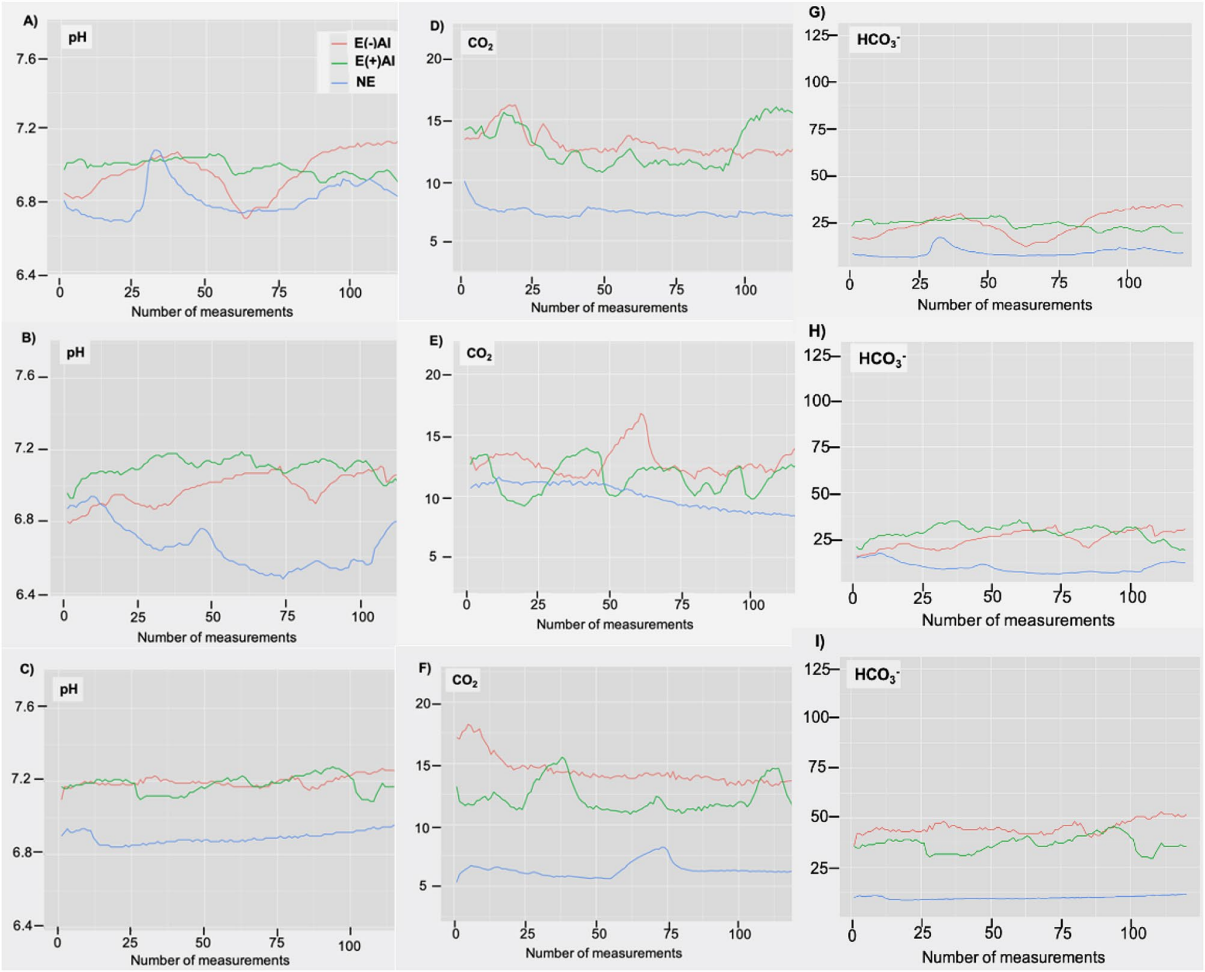
Plots of the timeline of uterine levels of pH **(A–C)**, CO_2_ (%) **(D–F)** and HCO_3_^−^ (mMol) **(G–I)** at the three experimental conditions in three selected sows. Corresponding figures for the same animal are horizontally displayed. Lines are a continuous representation of a set of 120 individual measurements (1 dot was recorded every 5 s for 10 min).

Individual plots of the timeline progress of all recorded pH values were represented (Fig. 1A–C). In some cases, the timeline depicted a waved pattern, with several variations higher than 0.2 pH units (Fig. 1A), while other displayed a quite a flat pattern with few and smooth waves (Fig. 1C). Characterization of flat and waved patterns was attempted depending on the number of waves higher than an established criterion, i.e. “a waved pattern displays > 2 waves of > 0.2 pH units”. However, as flat and waved patterns were found in animals of the three experimental conditions, and even within the same animal (i.e. Figure 1A, red and blue lines looked like a waved pattern while green line was rather flat) no statistical association between the defined pattern and the experimental conditions was found (Chi-squared test *p* > 0.05).

**Figure 2:**
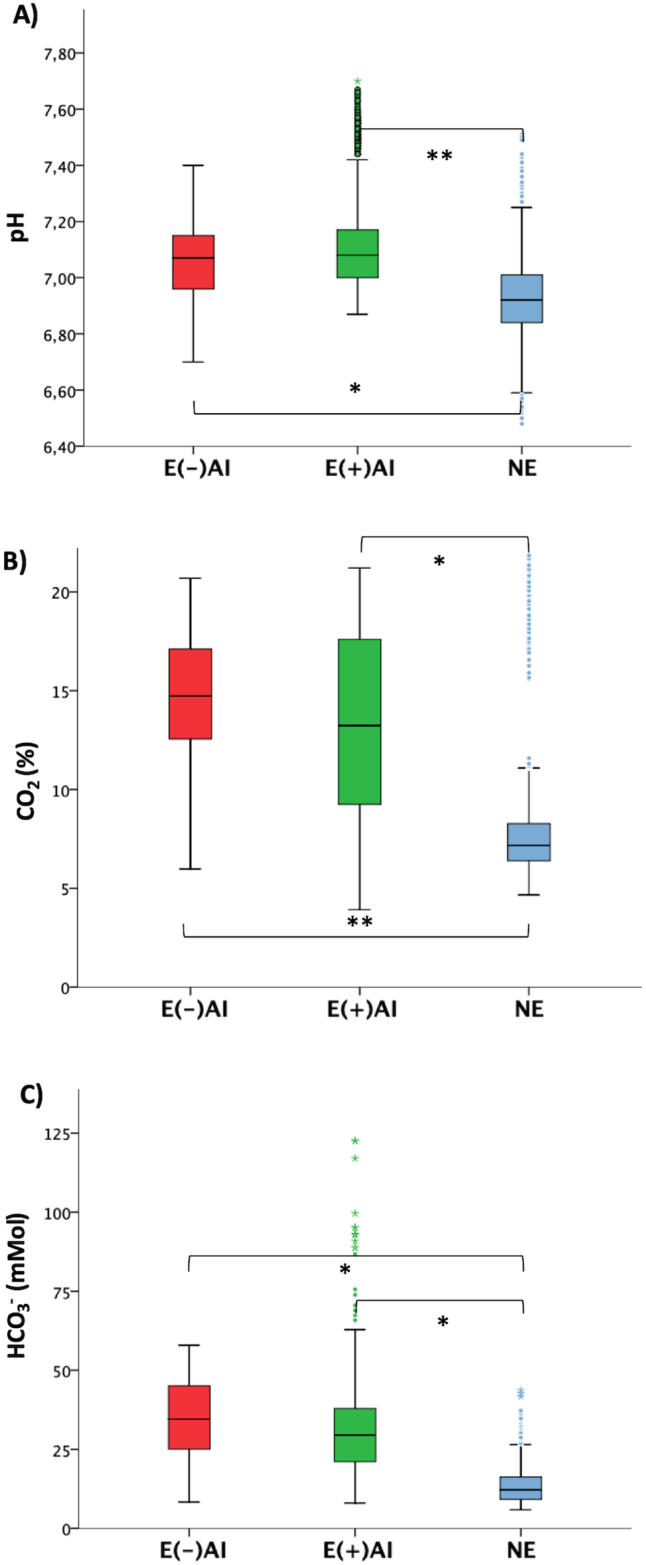
Box-plot describing the uterine levels of pH **(A)**, CO_2_ (%) **(B)** and HCO_3_^−^ (mMol) **(C)** at the three experimental conditions. In each panel, significant differences between experimental groups are indicated as *P < 0.05 and **P < 0.001. Small circles and asterisks in the box-plot represent outliers and extreme outliers, respectively.

A comparison of the uterine pH with statistical significance between the three experimental conditions is shown in Fig. 2A. An immediate effect of AI on the uterus pH was dismissed. Although the average pH in E(−)AI sows was a bit lower than in the E( +)AI group (0.06 units), such a small difference was not found to be significant (7.05 ± 0.13 and 7.11 ± 0.16, respectively, *p* > 0.05). However, uterine pH in sows of the NE group (6.93 ± 0.16) was significantly lower than in groups E(−)AI and E( +)AI (*p* = 0.017 and *p* = 0.007, respectively), revealing a significant increase of the uterus pH at estrous.

### CO_2_ (%) levels within the uterus during estrous (before and after insemination) and diestrous stages

Plots of the timeline for the recorded % of CO_2_ were obtained for each pig (Fig. 1D–F). As for the pH, the timeline variation of CO_2_% described arbitrary curves with a variable number of undulations, indistinctly of the experimental group and individual.

A by-group comparison of the data with statistical significance is displayed in Fig. 2B. The average % of uterine CO_2_ in E(-)AI group was 14.45 ± 3.58, and in E( +)AI group 13.12 ± 5.09. No effect of AI on the uterine % CO_2_ was observed (*p* > 0.05), so the presence of sperm in the uterus for 2 h hardly changed the % CO_2_. Conversely, the average uterine % CO_2_ at NE condition was 8.44 ± 3.71, representing a very strong decrease between estrous and diestrous stages (p _*NE vs E(−)AI*_ = 0.0002 and p _*NE vs E(*+*)AI*_ = 0.0019).

### HCO_3_^−^ concentration within the uterus in estrous (before and after insemination) and diestrous stages based on pH and CO_2_ activity

The individual time progress of the estimated concentration of HCO_3_^−^ (mMol) was also represented in individual plots (Fig. 1G–I). As it was the case of pH and CO_2_%, apparent flat or waved patterns were observed independently of the group and individual. As HCO_3_^−^ concentration was estimated by the Henderson-Hasselbalch equation with constant [CO_2_%] (average value in each pig), bicarbonate variations resembled the pH pattern of the same animal in the same experimental condition. The average concentration of HCO_3_^−^ (mMol) was 35.16 ± 11.79 and 30.99 ± 14.21 (mMol) for E( −)AI and E( +)AI conditions, respectively. So, as it was found for pH and CO_2_, AI did not change significantly the concentration of HCO_3_^−^ in the uterus (*p* > 0.05). Likewise, the average HCO_3_^−^ concentration in sows at NE condition was 14.94 ± 7.28 (mMol), which represented a high significant reduction with regards to the E( −)AI (*p* = 0.001) and E( +)AI (*p* = 0.003) (Fig. 2C).

## Discussion

The in vivo characterization of the physiological ambient within the female reproductive organs of mammals is important for both basic studies in the field of reproductive biology and ARTs. For this reason, the knowledge of the physical and chemical properties of the reproductive milieu by in situ estimation or after fluid collection from the oviduct, uterus and vagina has been largely attempted in humans, livestock and animal models^27,33^. With regards to the pH-CO_2_-HCO_3_^−^ biological regulator, very few studies have been addressed to the uterus (Table 1), which has only been approached in a few livestock species, but mainly cows. In pigs, only one work measured the uterine pH in vivo to a reduced number of animals via laparotomy^17^. In the present work, a more extensive study has been carried out and reference values of pH-CO_2_-HCO_3_^−^ levels in the porcine uterus have been obtained at two different stages of the estrous cycle and after insemination. Besides, to minimize iatrogenesis, all measurements were performed via a completely non-invasive cervical approach and without any medication. The combined use of a post-cervical AI catheter with miniaturized and semirigid pH and CO_2_ dipping probes allowed successful approaches to the uterine cavity (body and horns) in more than 90% of the attempts. Although the method did not give a visual evidence of the position of the sensors inside the uterine cavity, it was validated by both the perception of a smooth progression of the probes after having crossed the cervical canal (subjective), and the figures of insertion length of the probes (objective). The latest criterium was verified by comparing our results with reported figures of the length of the reproductive tract -from the *rima vulvae* to the uterine cavity- measured in *ex-vivo* organs of multiparous sows^34^. Our estimated insertion length for the pH and CO_2_ probes −64.2 ± 12.6 cm and 62 ± 11.5 cm, respectively- was higher than the reported post-mortem length of those organs (56.25 ± 6.01 cm). On the other hand, the total absence of medication or animal distress during the procedure guaranteed that the measurements were the closest to the physiological values, as it was also the case when a similar post-cervical approach was carried out for estimation of uterine pH in cows^20,21,25^.

Another novelty of the present work is the use of optical probes for the estimation of pH and CO_2_. Up to now, virtually all in vivo measurements of pH in the female reproductive organs were based on a miniaturized pH glass electrode combined with a reference electrode. While this technology proved useful and accurate for pH estimation, that was not the situation for CO_2_. Miniaturization of CO_2_ potentiometric sensors is technically more limited because a Severinghaus-type electrode is built by covering the pH microelectrode with a cap and a Teflon membrane in the tip. In addition to size restrictions for in vivo usage, the potentiometric CO_2_ sensors have scattered readings when immersed in complex fluid matrix such as the oviductal or uterine fluids (^16^; our experience with *unpublished results*). This most probably explains the lack of previous reports for CO_2_ estimation in vivo (Table 1). Unlike the potentiometric sensors, luminescent (optical) probes can be miniaturized to a few microns and display stable and accurate readings when immersed in complex biological matrix such as the reproductive fluids. This has already been proved for the determination of oxygen levels in vivo in reproductive organs of women and pigs^27,28^. For the first time, this work describes how pH and CO_2_ levels can be accurately measured in pigs with optical dipping probes directly inserted in the uterus. This is more relevant because from pH and CO_2_ levels, HCO_3_^−^ concentration was estimated, hence allowing a complete in vivo characterization of the bicarbonate buffer system. The relevance of characterizing this system for the optimization of ARTs protocols and the fact that the physical characteristics of the sensors (length, thickness, flexibility) can be customized under the manufacturer’s advice envisages an increasing usage of this technology in other organs and/or species.

From the best of our knowledge, this is the first description of CO_2_ and HCO_3_^−^ levels measured in situ in the uterus of animals. Only one attempt has been previously described in the oviduct of primates^16^. In the case of pH, the information is more extensive; several studies have reported in vivo values either in oviduct or uterus in different species (see Table 1). In sows in estrous the estimated uterine pH was 6.98^17^, values slightly lower than those presented here (7.05–7.11). This fact might be related to the use of halothane anesthesia in the early report, because this anesthetic causes a slight decrease of pH, not only in the blood but also in the oviduct^19^. Another remarkable result obtained from the experiments was that pH, CO_2_, and HCO_3_^−^ levels were higher during the estrous stage, independently of the insemination condition, than during diestrous. It is worth noting that the levels of reproductive hormones are quite different at estrous and diestrous stages. While the estrous is mainly dominated by estrogens, the situation changes during the first days of diestrous, when progesterone levels start to raise (reviewed by^35^). There are some evidences pointing that such hormonal changes have an impact on the pH-CO_2_-HCO_3_^−^ system. In rats, the expression of the different isoforms of carbonic anhydrase in the uterine tissue is directly regulated by the amount of estrogen and progesterone^36^, which suggests that the physiological uterine levels of pH–CO_2_–HCO_3_^−^ are dynamic and likely modified by the precise hormonal levels at each stage of the estrous cycle. In fact, exogenous estrogens administered in ovariectomized rats caused an increase in uterine fluid secretion and HCO_3_^−^ concentration^37^. More recently, it has been proposed a secretion/reabsorption activity for HCO_3_^−^ in the endometrial epithelium coordinated by progesterone and estrogen levels^38^. In estrous stage, when estrogen is dominant, HCO_3_^−^ secretion was up-regulated and reabsorption was down-regulated, and vice versa during diestrous stage, when progesterone is dominant. Nevertheless, the regulation of this complex system is far from being completely understood yet. For instance, different molecular mechanisms of the endometrial cells, such as the HCO_3_^−^/Cl^−^ and Na^+^/ HCO_3_^−^/Cl^-^ exchangers, and the Na^+^/H^+^ antiporter are likely involved in the pH and HCO_3_^−^ regulation^33^. Also, CFTR (cystic fibrosis transmembrane conductance regulator), which is responsible of numerous secretory responses, is an active regulator of the uterine secretion of HCO_3_^−^^9,39^. CFTR expression is enhanced in the uterus of females in early estrous but not in other stages^11^ where progesterone is enhanced^40,41^, suggesting a higher secretion of HCO_3_^−^ during estrous which is in accordance with our observations. Hydration of CO_2_ via carbonic anhydrase is another likely mechanism by which HCO_3_^−^ accumulates in the uterine fluid^14^. Likewise, in the case of pH our findings are in agreement with the literature, where a more alkaline uterine environment is necessary for sperm transport, capacitation and fertilization^39^ as occurs during the estrous stage, while an acidic pH, as observed in diestrous, is essential for embryo post-implantation development^42^.

Therefore, during estrous the uterine ambient is prepared to receive the spermatozoa, whose high levels of HCO_3_^−^ and alkaline environment, among other components, are necessary to initiate the sperm capacitation, a essential process for fertilization. It has been previously demonstrated that semen deposition induces an increase in carbonic anhydrase activity in endometrial tissues^43^ and causes a local shift in the gene expression of the female genital tract^44,45^, including some genes involved in pH regulation^46^. However, our results showed that the pH–CO_2_–HCO_3_^−^ system did not change after semen deposition within the uterus. Nevertheless, several factors could be involved in this fact. First, it has been shown that seminal plasma proteins and other components activated changes in the gene expression of the endometrium^45,47,48^; but in our study, the level of seminal plasma in the insemination dose was low because the ejaculate was diluted in commercial extender, so its impact on the genomic expression of the endometrium could be limited. Second, although uterus is colonized by the sperm within minutes after deposition^49^ the carbonic anhydrase activity in the endometrium is significantly increased after 4–6 h of sperm deposition^43^, and differences detected in gene expression were evaluated 24 h after semen deposition^44–46^; however, our measurements were carried out only 2 h after insemination. Thus, further studies with different time-points after insemination are necessary for a better understanding of the interactions between the uterine ambient and the sperm.

In vivo approaches to characterize the reproductive milieu such as the one in this study may have important benefits for the efficiency of the livestock industry and ARTs. Thus, the methodology presented here to estimate the levels of HCO_3_^−^ within the uterus might be useful for the detection of animals with impaired secretion of this relevant molecule with regards to their particular pH and CO_2_ values. Such alteration may result in compromised sperm capacitation, fertilization, or embryo development, as previously demonstrated in rodents^50,51^ and porcine^52^. So, having a criterion for assortment of sows with potential low fertility and/or prolificacy before entry to the reproductive system could be a relevant tool to optimizing the efficiency of the porcine industry^53^. Consequently, further studies should be directed to investigate this technology applied to field conditions and this study also contributes to this field. On the other hand, ARTs are commonly based on mimicking physiological conditions. As an example, when the physiological O_2_ tension found in the oviduct of sows was mimicked in the laboratory, both the final efficiency of embryo development and the quality increased compared to traditional conditions^28^. However, despite some clear evidences found in vitro, little attention has been paid to mimicking the in vivo sperm transit, specially through the uterine horns, for further use in ARTs. Thus, pH, CO_2_ or HCO_3_^−^ variations in culture media during in vitro gamete preparation and/or interaction showed relevant consequences in the fertilization output^54,55^. Also, an influence of the uterine fluid composition on sperm selection and consequent embryo culture was observed in vitro in several species including porcine^56–58^. But, in spite of these findings, the current laboratory conditions of boar sperm preparation prior to IVF are yet quite standard in the literature, using a temperature of 38.5 ºC, 5% CO_2_, 15–25 mMol of HCO_3_^−^ and ~ 7.2–7.4 pH. This is relevant because such conditions are far from those observed in our study at the estrous stage, not only in their particular figures (except the temperature) but also in the fact that they are not steady but rather dynamic or even oscillatory. Therefore, as sperm preparation methods should be as physiological as possible^32^, more in vitro studies are required to test how sperm manipulation under closest to real physiological conditions have an impact on the fertilization output and subsequent embryo development. From the results of the present study and those for oxygen and temperature^28,59^ the optimal conditions for sperm preparation to be tested would be, temperature 38.5ºC, pH 7.05–7.1, CO_2_ 13–14%, HCO_3_^−^ 30–35 mMol and O_2_ 7–10%.

## Conclusions

This study shows for the first time the combined values of pH, CO_2_, and HCO_3_^−^ in the uterus of the sow. By using a non-invasive approach and no medication during the procedure, precise reference values were obtained at different stages of the estrous cycle, and after insemination. The presence of sperm in the uterus hardly affected these parameters but were highly influenced by the estrous cycle, so that higher figures of pH, CO_2_, and HCO_3_^−^ were found at estrous than at non-estrous stage (diestrous). The study contributes to (i) a better understanding of the in vivo regulation of the pH/CO_2_/HCO_3_^−^ system in the uterus; (ii) get reference values of pH, CO_2_, and HCO_3_^−^ in a feasible physiological approach; (iii) obtain a new tool for tracking animals with impaired pH-CO_2_-HCO_3_^-^ system; (iv) a new set of parameters for the in vitro media where sperm are treated before IVF, mimicking the physiological ambient.

## Methods

### Animals and study design

The experiment was conducted on 12 sows [*Landrace x* commercial hybrids *(Large White* and *Duroc)* with 2.4 parities in average]. All animals showed normal reproductive performance previous to the experiments (total piglets born per litter 13.5 ± 1.0; live born per litter 10.9 ± 0.7). During the study, sows were housed in individual pens and fed by a commercial diet twice a day. Water was provided ad libitum. Environment was controlled by mechanical ventilation and evaporative cooling systems (temperature 22–24ºC). Multiparous sows were weaned 28 days after farrowing. Thenceforth, sows were observed twice daily for estrous behavior. Those exhibiting vulva reddening and swelling, and a standing reflex were considered in estrous.

**Figure 3:**
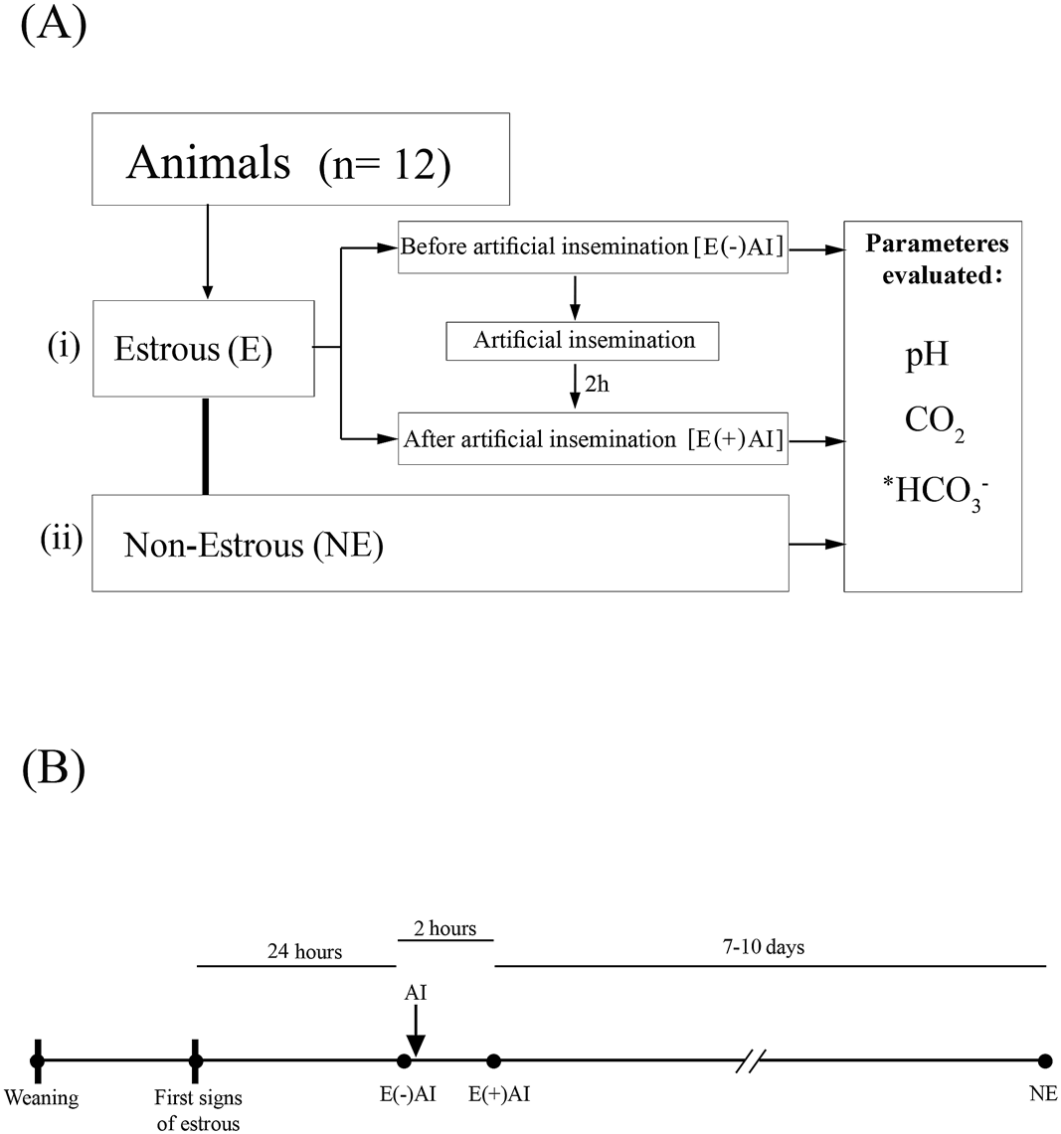
**(A)** Flow chart of the experimental design. *HCO_3_^−^ concentration was calculated from pH and CO_2_ data. (**B)** Timeline of experimental design. Black dots indicate the moment when pH and CO_2_ were measured.

pH and CO_2_ measurements were carried out at three different conditions (Fig. 3). The first set of measurements was done twenty-four hours after estrous detection [E( −)AI]. Immediately after this procedure, the females were artificially inseminated and 2 h later pH and CO_2_ measurements were repeated [E( +)AI]. For the insemination, a single dose of diluted semen was used. The inclusion criteria to use the seminal doses was total motility > 70%, viable sperm > 90% and morpho-abnomalies ≤ 15%. Post-cervical AI (seminal dose of 1.5 × 10^9^ sperm/45 ml) was performed with a disposable catheter (Soft&Quick, TecnoVet S.L., Spain)^60^. Finally, 7–10 days later, when the animals were at diestrous stage (non-estrous [NE]) the same procedure as at E( −)AI condition was repeated.

### Measurement of pH and CO_2_

**Figure 4:**
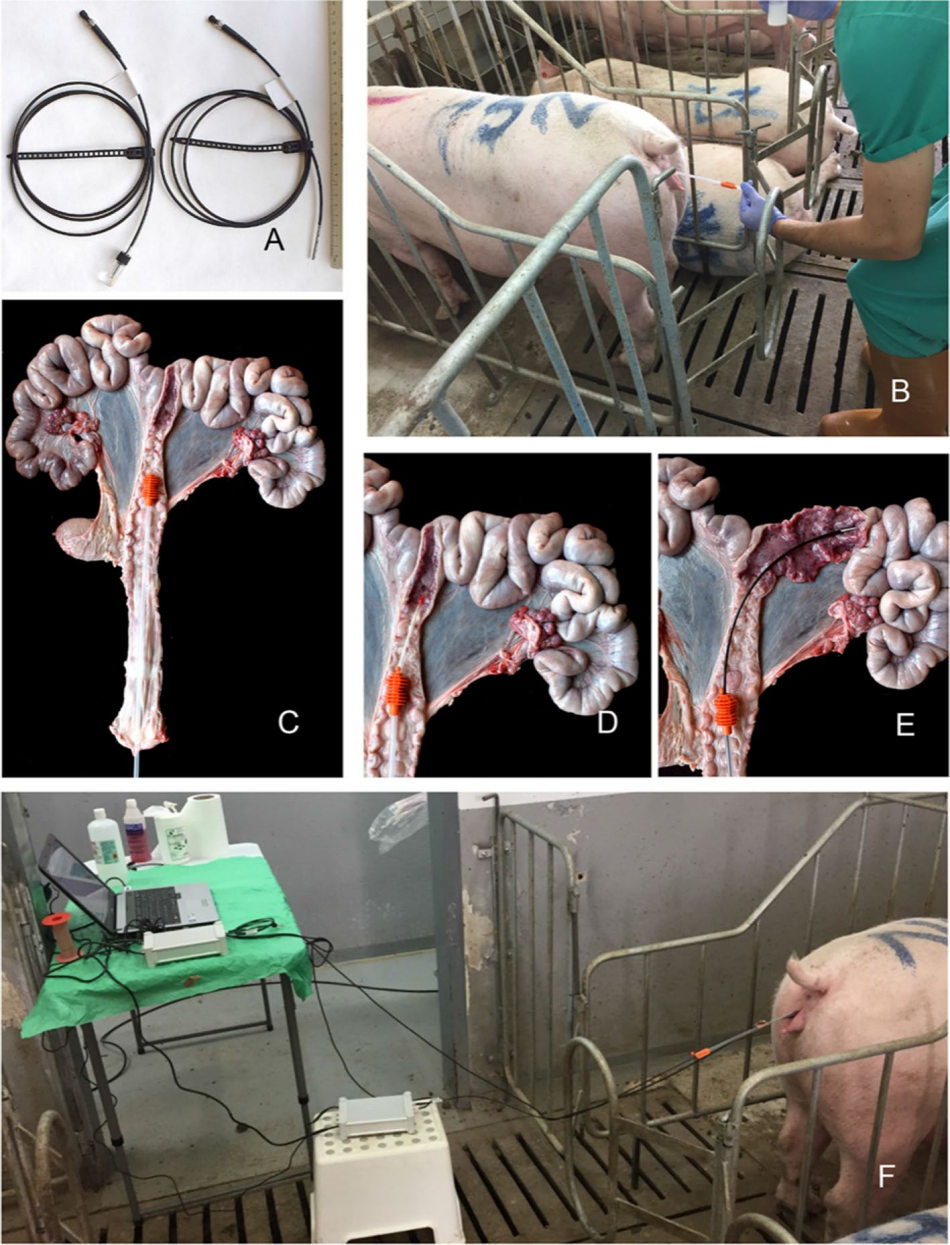
**(A)** Optical dipping probes of pH (right) and CO_2_ (left). **(B)** Insertion of disposable post-cervical AI catheter. **(C)** *Ex-vivo* genital tract simulating the estimated location of the AI catheter in the cervix. **(D)** Detail of “C” showing how the inner cannula of the AI catheter reaches the body of the uterus. **(E)** Detail in *ex-vivo* reproductive tract simulating how the pH probe is inserted within the uterus. After having passed through the AI catheter, the probe was pushed up to reaching the body and horns of the uterus. **(F)** Data recording from the uterus once the stabilization period is over.

pH and CO_2_ optical dipping probes -1500 mm long and 3 mm thick- were used (DP-HP5 and DP-CD1 prototypes, respectively, PreSens Precision Sensing GmbH—Am BioPark 11—93053 Regensburg, Germany) (Fig. 4A). Each probe was plugged into a recording unit (pH1 and PCO_2_ mini, PreSens Precision Sensing GmbH), which was connected to a laptop. Probes were pre-calibrated by the manufacturer, but calibration was also checked the day before use with standard pH 7 solution (Crison Instruments S.A., Spain), or 5% CO_2_ saturated saline solution (0.9%) within an embryo incubator. As pH and CO_2_ values are influenced by temperature, uterine temperature was always measured before inserting the optical probes. This was done with a 1500 mm long and 0.5 mm thick thermistor connected to an amplifier device (ThermaData Logger TCD; E.T.I. Electronic Temperature Instruments, West Sussex, UK).

At the three sampling conditions the following routine was followed. Briefly, a disposable post-cervical catheter (Soft&Quick, TecnoVet S.L., Spain) was used to facilitate the approach to the uterine lumen (body and horns) with the probes (Fig. 4B). When the catheter was positioned in the cervix (Fig. 4C), the inner cannula was gently pushed cranially to “help to open” the cervical lumen (Fig. 4D). The inner cannula was then removed and replaced by an endovascular catheter -900 mm long and 2.92 mm inner diameter- (reference 54–89,001, Terumo Europe), which allowed the temperature probe to reach the uterine cavity. Temperature was recorded for 10 min, and the stable temperature value used as a reference for pH and CO_2_ measurement in each animal. The endovascular catheter was then replaced by the pH probe, which was pushed cranially through the insemination catheter until no further progression was possible (Fig. 4E). When maximum insertion was reached, estimation of the insertion length within the reproductive tract was calculated by subtracting the total length of the probe to the distance from the *rima vulvae* to the recording unit. After a period of stabilization (2–5 min), and an indication to measure at the precise defined temperature, pH data were recorded every 5 s for a total of 10 min (Fig. 4F). The pH probe was then replaced by the CO_2_ probe, and the same procedure was followed for the recording of CO_2_ values. It is worth of mention that every time a probe was replaced the external opening of the insemination catheter was kept sealed to avoid the penetration of air within the cervix. All the protocol was done without any medication.

pH and CO_2_ data were exported into a worksheet (Excel, Microsoft) and used to estimate HCO_3_^−^ levels according to the Henderson-Hasselbalch equation, where pKa for a temperature of 38ºC is 6.11. As a simultaneous record of pH and CO_2_ was not available, the experimental average [CO_2_] in each animal was set as a constant value in the equation, and a point by point variation of [HCO_3_^−^] (mMol) was calculated depending on the pH.

$$\left[ {{\text{HCO}}_{3}^{ - } } \right] = \left[ {{\text{CO}}_{{2}} } \right]\times {10}^{{({{\rm pH}}{-}{{\rm pKa}})}}$$

### Statistical analysis

The statistical analysis was performed with the R program vs 3.4.4 (R Core Team 2018). Data of each experimental variable (pH, CO_2_, HCO_3_^−^) were explored by descriptive statistics and individual plots of the timeline of all the measurements displayed for each pig. ANOVA of repeated measures was carried out to evaluate potential differences between the three experimental conditions. Sphericity of data was always checked, and when required (as for the pH) corrected by Greenhouse–Geisser test. When ANOVA was significative between groups, comparison was done with post-hoc Tukey test. P values < 0.05 were considered statistically significant.

### Ethics approval and consent to participate

All the procedures carried out in this work were approved by the Ethical Committee of Animal Experimentation of the University of Murcia and by the Animal Production Service of the Agriculture Department of the Region of Murcia (Spain) (Ref. Nº. A13160606). Through the experiments, animals were handled carefully avoiding any unnecessary stress. All experiments were performed in accordance with relevant guidelines and regulations. The study was carried out in compliance with the ARRIVE guidelines (https://arriveguidelines.org/).

## Data Availability

All data analyzed during this study are included in this article. Raw data used to build graphs and plots are available upon request to the authors.

## References

[1] Buck J, Levin LR (2011). Physiological sensing of carbon dioxide/bicarbonate/pH via cyclic nucleotide signaling. Sensors.

[2] Swain JE (2012). Is there an optimal pH for culture media used in clinical IVF?. Hum. Reprod. Update.

[3] Okamura N, Tajima Y, Soejima A, Masuda H, Sugita Y (1985). Sodium bicarbonate in seminal plasma stimulates the motility of mammalian spermatozoa through direct activation of adenylate cyclase. J. Biol. Chem..

[4] Rodriguez-Martinez H, Ekstedt E, Einarsson S (1990). Acidification of epididymal fluid in the boar. Int. J. Androl..

[5] Hess KC (2005). The ‘soluble’ adenylyl cyclase in sperm mediates multiple signaling events required for fertilization. Dev. Cell.

[6] Austin C (1951). Observations on the penetration of the sperm in the mammalian egg. Aust. J. Sci. Res. B..

[7] Chang MC (1951). Fertilizing capacity of spermatozoa deposited into the fallopian tubes. Nature.

[8] Chan HC, Chen H, Ruan Y, Sun T (2012). Physiology and pathophysiology of the epithelial barrier of the female reproductive tract: role of ion channels. Adv Exp Med Biol.

[9] Zhou CX, Wang XF, Chan HC (2005). Bicarbonate secretion by the female reproductive tract and its impact on sperm fertilizing capacity. Sheng Li Xue Bao.

[10] Gholami K, Muniandy S, Salleh N (2013). Differential expression of Na+/H+-exchanger (NHE-1, 2, and 4) proteins and mRNA in rodent’s uterus under sex steroid effect and at different phases of the oestrous cycle. Biomed Res. Int..

[11] Chan LN (2002). Distribution and regulation of ENaC subunit and CFTR mRNA expression in murine female reproductive tract. J. Membr. Biol..

[12] He Q, Chen H, Wong CHY, Tsang LL, Chan HG (2010). Regulatory mechanism underlying cyclic changes in mouse uterine bicarbonate secretion: role of estrogen. Reproduction.

[13] Gholami K, Muniandy S, Salleh N (2014). Modulation of sodium-bicarbonate co-transporter (SLC4A4/NBCe1) protein and mRNA expression in rat’s uteri by sex-steroids and at different phases of the oestrous cycle. Res. Vet. Sci..

[14] Vishwakarma P (1962). The pH and bicarbonate-ion content of the oviduct and uterine fluids. Fertil. Steril..

[15] Rodriguez-Martinez H (2007). Role of the oviduct in sperm capacitation. Theriogenology.

[16] Maas DHA, Storey BT, Mastroianni L (1977). Hydrogen ion and carbon dioxide content of the oviductal fluid of the rhesus monkey (Macaca mulatta). Fertil. Steril..

[17] Mather, E C. Day, B. In vivo pH values of the estrous reproductive tract of the gilt. *Theriogenology* **8**, 323–327 (1977).

[18] Nichol R, Hunter RHF, Cooke GM (1997). Oviduct fluid pH in intact and unilaterally ovariectomized pigs. Can. J. Physiol. Pharmacol..

[19] Hugentobler S, Morris DG, Kane MT, Sreenan JM (2004). In situ oviduct and uterine pH in cattle. Theriogenology.

[20] Elrod CC, Butler WR (1993). Reduction of fertility and alteration of uterine pH in heifers fed excess ruminally degradable protein. J. Anim. Sci..

[21] Elrod CC, Van Amburgh M, Butler WR (1993). Alterations of pH in response to increased dietary protein in cattle are unique to the uterus. J. Anim. Sci..

[22] Penrod L, Deaver E, Erin K, Glenn C, Michelle L, Mark J (2011). Characterization of uterine pH during the estrous cycle of the mare. J Eq Vet Sci.

[23] Mather EC (1975). ‘In vivo’ uterine lumen pH values of the bovine. Theriogenology.

[24] Maas DH, Stein B, Metzger H (1984). PO2 and pH measurements within the rabbit oviduct following tubal microsurgery: reanastomosis of previously dissected tubes. Adv. Exp. Med. Biol..

[25] Kane KK (2002). Effects of varying levels of undegradable intake protein on endocrine and metabolic function of young post-partum beef cows. Theriogenology.

[26] Wencel D (2018). Optical sensor for real-time pH monitoring in human tissue. Small.

[27] Ottosen LDM (2006). Observations on intrauterine oxygen tension measured by fibre-optic microsensors. Reprod. Biomed. Online.

[28] García-Martínez S (2018). Mimicking physiological O2 tension in the female reproductive tract improves assisted reproduction outcomes in pig. Mol. Hum. Reprod..

[29] Schachtschneider KM (2015). Adult porcine genome-wide DNA methylation patterns support pigs as a biomedical model. BMC Genomics.

[30] Lorenzen, E., Follmann, F., Jungersen, G. & Agerholm, J. S. A review of the human vs. porcine female genital tract and associated immune system in the perspective of using minipigs as a model of human genital Chlamydia infection. *Vet. Res.* **46**, 1–13 (2015). 10.1186/s13567-015-0241-9 PMC4586017 26411309

[31] Fowler KE, Mandawala AA, Griffin DK, Walling GA, Harvey SC (2018). The production of pig preimplantation embryos in vitro: Current progress and future prospects. Reprod. Biol..

[32] Romar R, Cánovas S, Matás C, Gadea J, Coy P (2019). Pig in vitro fertilization: where are we and where do we go?. Theriogenology.

[33] Ng KYB, Mingels R, Morgan H, Macklon N, Cheong Y (2018). In vivo oxygen, temperature and pH dynamics in the female reproductive tract and their importance in human conception: a systematic review. Hum. Reprod. Update.

[34] García-Vázquez, F. A., Llamas-López, P. J., Jacome, M. A., Sarrias-Gil, L. & López Albors, O. Morphological changes in the porcine cervix: A comparison between nulliparous and multiparous sows with regard to post-cervical artificial insemination. *Theriogenology* **127**, 120–129 (2019). 10.1016/j.theriogenology.2019.01.004 30685687

[35] Soede NM, Langendijk P, Kemp B (2011). Reproductive cycles in pigs. Anim. Reprod. Sci..

[36] Karim K, Giribabu N, Muniandy S, Salleh N (2016). Estrogen and progesterone differentially regulate carbonic anhydrase II, III, IX, XII, and XIII in ovariectomized rat uteri. Syst. Biol. Reprod. Med..

[37] Shahzad, H. *et al.* Combinatorial effects of quercetin and sex-steroids on fluid and electrolytes’ (Na+, Cl-, HCO3-) secretory mechanisms in the uterus of ovariectomised female Sprague-Dawley rats. *PLoS One* **12**, (2017). 10.1371/journal.pone.0172765 PMC5333842 28253299

[38] Xie, Z. D. *et al.* The balance of HCO3-Secretion vs. reabsorption in the endometrial epithelium regulates uterine fluid pH. *Front. Physiol.* **9**, 12 (2018). 10.3389/fphys.2018.00012 PMC5788990 29422866

[39] Liu Y, Wang D-K, Chen L-M (2012). The physiology of bicarbonate transporters in Mammalian. Biol. Reprod..

[40] Mularoni A, Beck L, Sadir R, Adessi GL, Nicollier M (1995). Down-regulation by progesterone of CFTR expression in endometrial epithelial cells: a study by competitive RT-PCR. Biochem. Biophys. Res. Commun..

[41] Yang JZ (2004). Differential expression and localization of CFTR and ENaC in mouse endometrium during pre-implantation. Cell Biol. Int..

[42] Sun-Wada GH (2000). Acidic endomembrane organelles are required for mouse postimplantation development. Dev. Biol..

[43] Collado ML, Castro G, Hicks JJ (1979). Effect of spermatozoa upon carbonic anhydrase activity of rabbit endometrium. Biol. Reprod..

[44] López-Úbeda R (2015). Oviductal transcriptome is modified after insemination during spontaneous ovulation in the sow. PLoS ONE.

[45] Álvarez-Rodríguez M, Martinez CA, Wright D, Rodríguez-Martinez H (2020). The role of semen and seminal plasma in inducing large-scale genomic changes in the female porcine peri-ovulatory tract. Sci. Rep..

[46] Atikuzzaman M, Bhai RM, Fogelholm J, Wright D, Rodriguez-Martinez H (2015). Mating induces the expression of immune- and pH-regulatory genes in the utero-vaginal junction containing mucosal sperm-storage tubuli of hens. Reproduction.

[47] Chen JC (2014). Seminal plasma induces global transcriptomic changes associated with cell migration, proliferation and viability in endometrial epithelial cells and stromal fibroblasts. Hum. Reprod..

[48] Martinez CA (2019). Seminal plasma modifies the transcriptional pattern of the endometrium and advances embryo development in pigs. Front. Vet. Sci..

[49] Hunter RH (1981). Sperm transport and reservoirs in the pig oviduct in relation to the time of ovulation. J. Reprod. Fertil..

[50] Wang XF (2003). Involvement of CFTR in uterine bicarbonate secretion and the fertilizing capacity of sperm. Nat. Cell Biol..

[51] Muchekehu RW, Quinton PM (2010). A new role for bicarbonate secretion in cervico-uterine mucus release. J. Physiol..

[52] Chen MH (2010). Involvement of CFTR in oviductal HCO-3 secretion and its effect on soluble adenylate cyclase-dependent early embryo development. Hum. Reprod..

[53] García-Vázquez FA (2019). Post-cervical artificial insemination in porcine: the technique that came to stay. Theriogenology.

[54] Soriano-Úbeda C, García-Vázquez FA, Romero-Aguirregomezcorta J, Matás C (2017). Improving porcine in vitro fertilization output by simulating the oviductal environment. Sci. Rep..

[55] Soriano-Úbeda, C., Romero-Aguirregomezcorta, J., Matás, C., Visconti, P. E. & García-Vázquez, F. A. Manipulation of bicarbonate concentration in sperm capacitation media improvesin vitro fertilisation output in porcine species. *J. Anim. Sci. Biotechnol.* **10**, (2019). 10.1186/s40104-019-0324-y PMC6410524 30899459

[56] Kawano N (2014). Seminal vesicle protein SVS2 is required for sperm survival in the uterus. Proc. Natl. Acad. Sci. USA..

[57] Canovas S (2017). DNA methylation and gene expression changes derived from assisted reproductive technologies can be decreased by reproductive fluids. Elife.

[58] Luongo C, Abril-Sánchez S, Hernández JG, García-Vázquez FA (2019). Seminal plasma mitigates the adverse effect of uterine fluid on boar spermatozoa. Theriogenology.

[59] García-Martínez S, Latorre R, Sánchez-Hurtado MA, Sánchez-Margallo FM, Bernabo N, Romar R, López-Albors O, C. P. Mimicking the temperature gradient between the sow’s oviduct and uterus improves in vitro embryo culture output. *Mol. Hum. Reprod.* (2020). 10.1093/molehr/gaaa053 32647896

[60] Hernández-Caravaca I (2012). Reproductive performance and backflow study in cervical and post-cervical artificial insemination in sows. Anim. Reprod. Sci..

